# Reduced expression of Rho GDP dissociation inhibitor 2 mRNA is associated with lymph node metastasis in gastric carcinoma

**DOI:** 10.3892/ol.2013.1379

**Published:** 2013-06-06

**Authors:** ATSUO SHIDA, SHUICHI FUJIOKA, NAOTO TAKAHASHI, HIROAKI AOKI, TOMOYOSHI OKAMOTO, NORIO MITSUMORI, NOBUO OMURA, KATSUHIKO YANAGA

**Affiliations:** Department of Surgery, Jikei University School of Medicine, Minato-ku, Tokyo 105-8461, Japan

**Keywords:** Rho GDP dissociation inhibitor 2, gastric carcinoma

## Abstract

Small GTPase proteins, including RhoA, RhoB, RhoC, Rac1 and cdc42, are molecules that have significant roles in linking cell shape and cell cycle progression in cytoskeletal arrangements and mitogenic signaling. Rho GDP dissociation inhibitor 2 (RhoGDI2) has recently been identified as a metastasis suppressor gene in models of bladder cancer. RhoGDI2 has also been identified as a potential regulator of tumorigenesis and cancer progression. The present study aimed to clarify the significance of RhoGDI2 gene expression in gastric carcinoma and to evaluate the outcome of affected patients. A total of 46 pairs of normal mucosa and cancer specimens were obtained from patients who had undergone a gastrectomy for primary gastric carcinoma and were subjected to semi-quantitative reverse transcription polymerase chain reaction (RT-PCR) for RhoGDI2. The expression of RhoGDI2 mRNA was significantly higher in early-stage gastric cancer specimens compared with the normal gastric epithelium samples. By contrast, the depth of the tumor was negatively correlated with RhoGDI2 mRNA expression. In addition, a reduced expression of RhoGDI2 mRNA was associated with venous system invasion and lymph node metastasis. RhoGDI2 mRNA was more frequently expressed in differentiated adenocarcinoma compared with poorly-differentiated adenocarcinoma. Although the statistical significance was not established, RhoGDI2-positive patients tended to have a superior oncological outcome compared with RhoGDI2-negative patients. The reduced expression of RhoGDI2 mRNA in gastric carcinoma is associated with venous system invasion and lymph node metastasis.

## Introduction

The Rho GTPases, which are from a distinct branch of the Ras-like low molecular weight GTP-binding protein super-family, are involved in actin cytoskeleton organization (1) and have been associated with invasion and metastasis (2). RhoGTPases alternate between an inactive GDP-bound state and an active GTP-bound state. The regulators of this GDP/GTP cycle include GDP dissociation inhibitors (GDIs), which bind to the inactive form, thereby blocking further activation. RhoGDI1 was first identified on the basis of its ability to inhibit GDP dissociation from RhoA (3), CDC42Hs (4) and Rac1 (5). RhoGDI2, also known as D4-GDI or Ly-GDI, shares a 67% amino acid analogy with RhoGDI1 (6–8). However, in contrast with the ubiquitous property of RhoGDI1, the protein is believed to be expressed exclusively on cells of hematopoietic lineage (6,7). However, Seraj *et al* (9) and Gildea *et al* (10) suggested that the RhoGDI2 gene is also expressed in non-hematopoietic neoplasms. Furthermore, Gildea *et al* (11) have shown using an animal model of human bladder cancer metastasis and DNA microarray technology that RhoGDI2 is a putative metastasis suppressor gene in human cancer. By analyzing patient immunohistochemistry (IHC), Theodorescu *et al* reported that RhoGDI2 is an independent predictor of prognosis for patients with bladder cancer (12). Also, Hu *et al* (13) demonstrated that the reduced expression of RhoGDI2 in breast cancer was associated with lymph node metastasis. To date, there have been no studies on the impact of RhoGDI2 mRNA expression for the survival of patients with gastric carcinoma. Hence, the present study investigated the expression of RhoGDI2 in human gastric cancer specimens and evaluated the significance of RhoGDI2 expression on the outcome and clinicopathological parameters of the patients.

## Patients and methods

### 

#### Patients and tumor samples

A total of 46 patients who had undergone a gastrectomy with lymph node dissection for primary gastric carcinoma at Jikei University Daisan Hospital (Tokyo, Japan) and Shiomidai Prefectural Hospital (Kanagawa, Japan) between March 2006 and January 2010 were studied. Pre-operative informed consent was obtained from each patient in accordance with institutional guidance. Tumor specimens and normal mucosal tissues were stored at 4°C in an RNA preserving reagent (RNA-later, Ambion, Austin, TX, USA). The extraction of total RNA from the samples was performed within three months of tissue extraction. The total RNA was immediately transcribed to first-strand cDNA (1st Strand cDNA Synthesis kit; Roche, Basel, Switzerland), which was stored at −80°C until the reverse transcription-polymerase chain reaction (RT-PCR). RT-PCR for RhoGDI2 and glyceraldehyde 3-phosphate dehydrogenase (GAPDH) was performed between July 2011 and August 2011. The RhoGDI2 and GAPDH mRNA levels were quantified using NIH Image 1.63 computer software (Wayne Rasband, NIH, Bethesda, MD, USA), and the intensity of RhoGDI2 for GAPDH was calculated. Pathological and clinical records were reviewed and the disease stage was determined according to the classification by the Japanese Research Society for gastric cancer (14). The histological grading and depth of invasion of the tumors in all the cases were available and are summarized in Table I. The presence of liver metastasis and peritoneal dissemination was determined pre-operatively using radiographic examinations or intra-operatively. Based on the histological grade, the tumor specimens were classified into two groups consisting of the differentiated group (well- to moderately-differentiated adenocarcinoma and papillary adenocarcinoma) and the undifferentiated group (poorly-differentiated adenocarcinoma and signet-ring cell or mucinous carcinoma). This study was approved by the ethics committee of Jikei University School of Medicine, Tokyo, Japan.

#### Semi-quantitative RT-PCR

Total RNA was extracted from the resected gastric carcinoma and normal tissue specimens using the RNeasy Mini kit (Qiagen, Valencia, CA, USA), according to the manufacturer’s instructions. The first-strand cDNA was synthesized from total RNA by reverse transcriptase (1st Strand cDNA Synthesis kit; Roche, Basel, Switzerland). The PCR analysis was performed using the following pairs of primers: RhoGDI2 forward, 5′-agtacgacgtgatcgtgctg-3′ and reverse, 5′-gcgagcaatttctccttcag-3′; and GAPDH forward, 5′-atcatccctgcctctactgg-3′ and reverse, 5′-ccctccgacgcctgcttcac-3′. Following this, 1 *μ*g/l of the cDNA reaction was subjected to 35 PCR cycles (denaturing at 94°C for 1 min, annealing at 58°C for 50 sec and polymerization at 72°C for 1 min), in the presence of 0.25 U Taq DNA Polymerase (Roche, Indianapolis, IN, USA), 1X PCR reaction buffer (Roche), 0.25 mM dNTPs (Promega, Madison, WI, USA) and 0.5 *μ*M specific primers for RhoGDI2 and GAPDH in a final reaction volume of 50 *μ*l.

#### IHC

Using paraffin-embedded specimens from eight patients with gastric cancer, the RhoGDI2 protein was detected using the anti-RhoGDI2 rabbit polyclonal antibody (Abcam, Cambridge, UK). Briefly, subsequent to being microwaved in citrate buffer solution (pH 6.0), the deparaffinized sections were incubated with 1% methanol-hydrogen peroxide for 30 min. The slides were then incubated with the rabbit polyclonal antibody against RhoGDI2 (undiluted solution) for 60 min. This was followed by incubation with anti-rabbit secondary antibody (Envision™/Rabbit/HRP; Dako, Carpinteria, CA, USA) for 30 min. The staining was visualized using the diaminobenzidine (DAB) method (Dako) for 5 min. Counter-staining was performed lightly with hematoxylin. All incubations were performed at room temperature in a humidified chamber. Control rabbit immunoglobulin G was used for each staining (Daiichi Fine Chemical, Takaoka, Toyama, Japan).

#### Statistics

The significance of the data was determined using the chi-square test or Student’s t-test. The multivariate analysis for patient prognosis was determined using the Cox proportional hazards model. The survival curves of the patients were compared using the Kaplan-Meier method and analyzed by the log-rank test. P<0.05 was considered to indicate a statistically significant difference.

## Results

### Expression of RhoGDI2 mRNA in human gastric carcinoma tissues

#### Semi-quantitative RT-PCR

**Figure 1.**
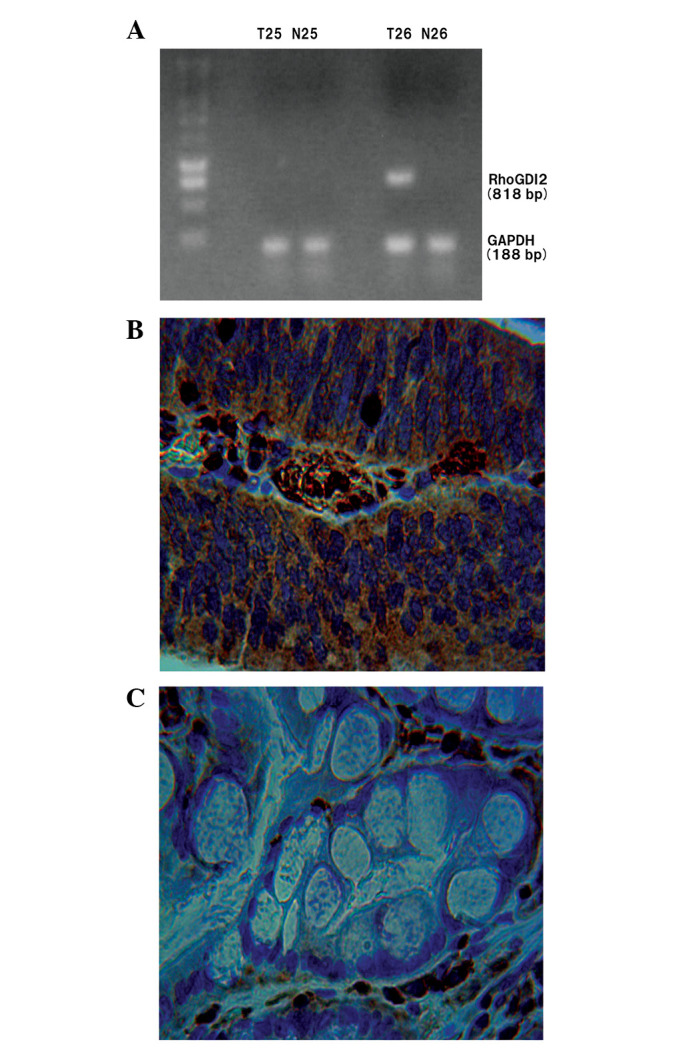
(A) RT-PCR analysis of RhoGDI2 and GAPDH mRNA in two human gastric carcinoma patients. In patient 25, RhoGDI2 mRNA was not expressed in the T or N mucosa. However, in patient 26, RhoGDI2 mRNA was expressed in the T but not the N mucosa. (B) Representative IHC staining with RhoGDI2 polyclonal antibody in gastric cancer, showing strong staining for RhoGDI2 in the cytoplasm of the tumor tissue (×400). (C) In the N gastric mucosa, RhoGDI2 was not observed in the epithelial cells (×400). The samples shown in (B) and (C) are derived from patient 26. RT-PCR, reverse transcription-polymerase chain reaction; RhoGDI2, Rho GDP dissociation inhibitor 2; GAPDH, glyceraldehyde-phosphate dehydrogenase; T, tumor; N, normal; IHC, immunohistochemistry.

A total of 46 pairs of tissue samples obtained from the tumors of patients with gastric carcinoma and the adjacent non-cancerous mucosa were examined for RhoGDI2 gene expression using RT-PCR (Fig. 1).

**Figure 2.**
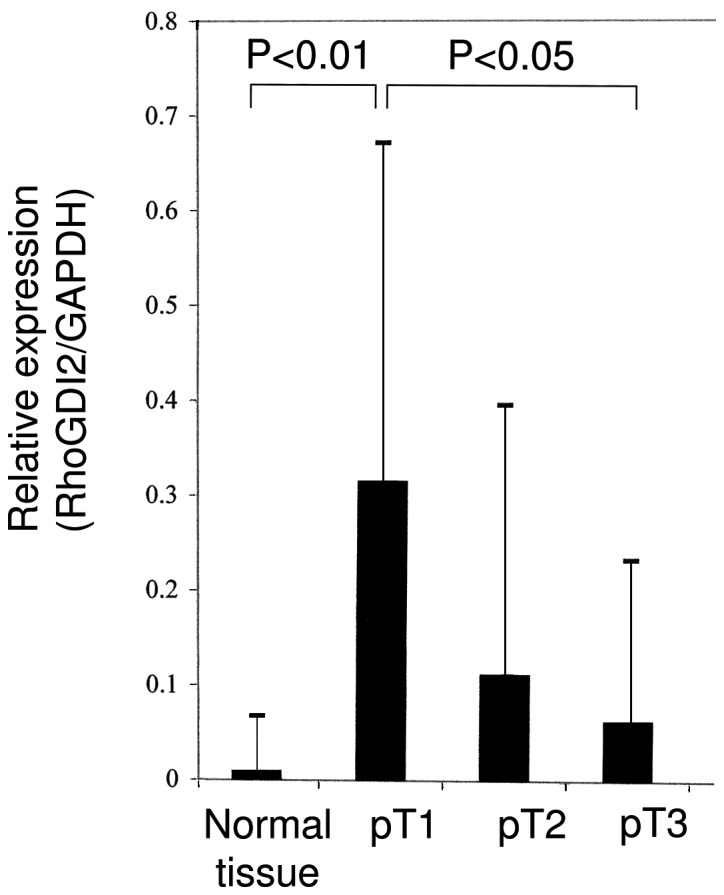
Semi-quantitative RT-PCR analysis of RhoGDI2 mRNA. The relative intensity of RhoGDI2 for GAPDH was estimated with computer software (NIH Image 1.63, Wayne Rasband, NIH). The relative expression of RhoGDI2 was compared using the Student’s t-test. RhoGDI2 mRNA expression in early gastric cancer was significantly higher compared with normal mucosa and serosa exposed advanced gastric cancer. RhoGDI2, Rho GDP dissociation inhibitor 2; GAPDH, glyceraldehyde 3-phosphate dehydrogenase; RT-PCR, reverse transcription-polymerase chain reaction.

**Figure 3.**
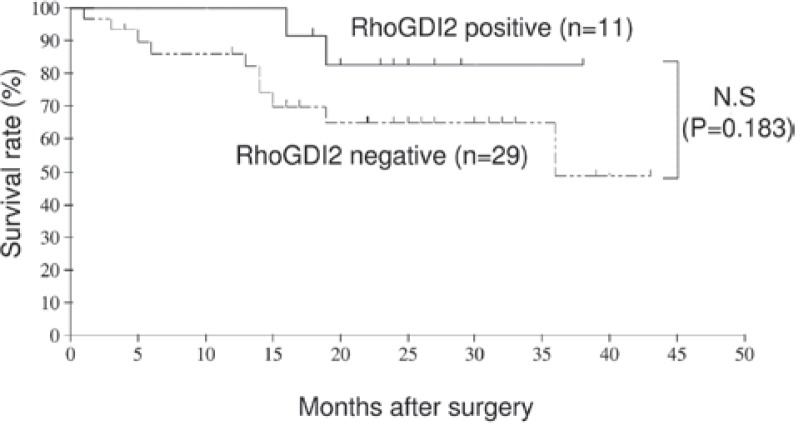
Post-operative relapse-free survival (RFS) of patients with or without RhoGDI2 mRNA expression in gastric carcinoma. RhoGDI2, Rho GDP dissociation inhibitor 2; N.S, not significant.

**Figure 4.**
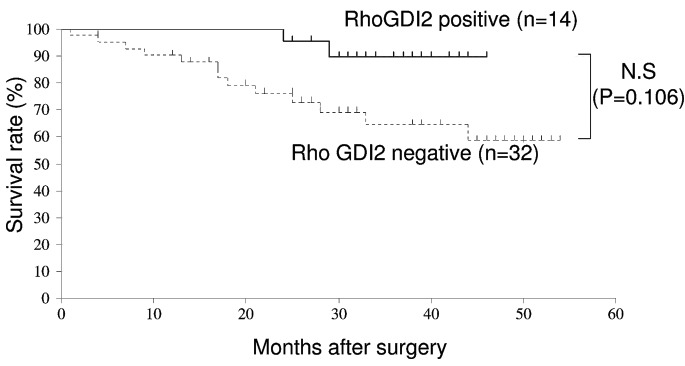
Post-operative overall survival (OS) of patients with or without RhoGDI2 mRNA expression in gastric carcinoma. RhoGDI2, Rho GDP dissociation inhibitor 2; N.S, not significant.

The 95% CI [the average ratio + two standard deviations (SD)] was used to select a cut-off line. The mean intensity of RhoGDI2 for GAPDH in 46 normal gastric tissues was 0.01 and the SD was 0.057. Hence, the cut-off value of RhoGDI2 was defined as 0.124. According to this cut-off line, RhoGDI2-positive expression was observed in 14 (30.4%) human gastric carcinoma samples and in one (2.2%) normal gastric mucosa sample. The expression of RhoGDI2 mRNA was significantly higher in the early-stage gastric cancer samples compared with the normal gastric mucosa or advanced gastric cancer tissues (Fig. 2; Student’s t-test, P<0.01). A univariate analysis was carried out to determine the correlation between the conventional pathological prognostic markers and RhoGDI2 mRNA expression. The results show that a reduced expression of RhoGDI2 is associated with venous system invasion and lymph node metastasis (Table II). The tumor expression of RhoGDI2 was further evaluated as a prognostic variable in patients with gastric carcinoma. Table III and IV show the multivariate analysis, which identified the fact that RhoGDI2 expression was not an independent prognostic factor for relapse-free survival (RFS) or overall survival (OS). However, the data show that RhoGDI2-positive patients had a good prognosis compared with those who were RhoGDI2-negative (Figs. 3 and 4).

#### IHC

The expression of the RhoGDI2 protein was assessed in eight cases of surgically removed gastric tissues using a polyclonal antibody that was specific to RhoGDI2. In several cases, strong staining for RhoGDI2 was observed in the cytoplasm of the tumor tissues, even though negative or very weak RhoGDI2 was observed in the normal gastric tissues.

These IHC examinations corresponded with the results from the RT-PCR (Fig. 1).

## Discussion

RhoGDIs are known to inhibit the activation of RhoGTPases (15). RhoGDI2 has been established as a metastasis suppressor gene in bladder cancer. Using a gene array analysis, Theodorescu *et al* reported that decreased RhoGDI2 gene/protein expression was associated with a more invasive variant of the HRAS mutation-positive T24 bladder cancer cell line (12). Ectopic restoration of RhoGDI2 expression in the invasive T24 variant has also been shown to decrease metastasis, as determined by tail-vein lung tumor colonization in mice (10). Finally, the IHC analysis of 51 bladder tumors revealed that RhoGDI2 overexpression correlated with a poor survival time to disease-specific mortality (12). Hu *et al* identified a biphasic pattern of increased RhoGDI2 expression with breast hyperplasia, but decreased expression with progression and lymph node metastasis using IHC staining in 71 patients (13). In addition, Stevens *et al* showed that a stable suppression of RhoGDI2 protein expression in ovarian cancer cells increased anchorage-independent growth and Matrigel invasion *in vitro,* and in tail-vein lung colony metastatic growth *in vivo* (16). These data show that RhoGDI2 suppresses cancer progression.

Although an increasing number of studies on the role of RhoGDI2 are appearing, the function of RhoGDI2 remains controversial. Tapper *et al* demonstrated that the upregulation of RhoGDI2 was associated with the malignant potential of ovarian carcinoma by cDNA array analysis (17). An increased motility of murine cancer has been reported to correlate with an overexpression of RhoGDI2 (18). In addition, Cho *et al* observed that the ectopic overexpression of RhoGDI2 in poorly-invasive gastric carcinoma cell lines significantly increased Matrigel invasiveness *in vitro*. Conversely, the depletion of endogenous RhoGDI2 in RhoGDI2-overexpressing gastric carcinoma cells suppressed invasion *in vitro*. A forced expression of RhoGDI2 in these cells lines increased tumor growth, angiogenesis and lung metastasis in mice (19). The findings indicate that RhoGDI2 is involved in tumorigenesis and cancer progression.

According to the present data, the reduced expression of RhoGDI2 is associated with venous system invasion and lymph node metastasis. The data suggest that RhoGDI2 suppresses cancer metastasis and that the detection of RhoGDI2 mRNA expression may be an useful clinical modality for detecting lymph node metastasis in gastric carcinoma. Dransart *et al* (20) and Stevens *et al* (16) found that RhoGDI2 preferentially bound and activated Rac1, and that Rac1 activation antagonized metastasis. Since RhoGDIs are able to modulate Rho GTPase interaction with Rho guanine nucleotide exchange factor (RhoGEFs) and Rho GTPase-activating proteins (RhoGAPs), RhoGDI2 may activate Rac through the altered regulation of GDP/GTP cycling and then Rac activation may be able to antagonize tumor invasion and metastasis (15).

In summary, the RhoGDI2 mRNA expression that is usually decreased in advanced-stage gastric cancer was significantly increased in the early-stage gastric cancer patients of the present study. Such expression was almost non-existent in the normal epithelial samples. A reduced expression of RhoGDI2 is associated with venous system invasion and lymph node metastasis, which may lead to the formation of clinical applications for the evaluation of lymph node metastasis in patients with gastric carcinoma.

## Figures and Tables

**Table I.** Clinical and pathological characteristics of the patients and tumors.

Characteristic	Value
Median age, years	73
Gender, n (male:female)	31:15
Median size, mm	40
Histological type, n (%)	
Differentiated	27 (59)
Undifferentiated	19 (41)
Location of tumor, n (%)	
U	10 (22)
Mid	25 (54)
L	11 (24)
Gross form, n (%)	
0-I	1 (2)
0-IIa	7 (15)
0-IIb	1 (2)
0-IIc	8 (17)
0-III	0 (0)
Type 1	2 (4)
Type 2	12 (26)
Type 3	14 (30)
Type 4	1 (2)
Depth of invasion, n (%)	
pT1 (M, SM)	15 (32)
pT2 (MP, SS)	20 (43)
pT3 (SE)	11 (24)
pT4 (SI)	0 (0)
Venous invasion^+^, n (%)	20 (43)
Lymphatic invasion^+^, n (%)	36 (78)
Lymph node metastasis^+^, n (%)	25 (54)
Peritoneal dissemination^+^, n (%)	3 (7)
Synchronous liver metastasis^+^, n (%)	1 (2)
Synchronous lung metastasis^+^, n (%)	0 (0)

U, upper; Mid, middle; L, lower; pT, pathological tumor depth; M, mucosa; SM, submucosa; MP, muscularis propia; SS, subserosa; SE, serosa exposed; SI, other organ invasion.

**Table II.** Correlation between clinicopathological observations and RhoGDI2 expression.

Findings	RhoGDI2 expression		P-value
	Positive, n	Negative, n	
Histological type			
Differentiated	11	16	
Undifferentiated	3	16	0.1374
Location of tumor			
U	3	7	
M	10	15	
L	1	10	0.1555
Gross form			
0-I	1	0	
0-IIa	3	4	
0-IIb	1	0	
0-IIc	3	5	
0-III	0	0	
Type 1	2	0	
Type 2	2	10	
Type 3	2	12	
Type 4	0	1	0.1214
Venous invasion			
Positive	3	19	
Negative	11	13	0.0404
Lymphatic invasion			
Positive	8	22	
Negative	6	10	0.4469
Lymph node metastasis			
Positive	3	23	
Negative	11	9	0.0043

U, upper; M, middle; L, lower; RhoGDPI2, Rho GDP dissociation inhibitor 2.

**Table III.** Risk factors affecting RFS as determined by the Cox proportional hazards model in 40 patients with gastric cancer.

Variable	Multivariate analysis for RFS		
	Hazard ratio	95% CI	P-value
pT (T1 vs. T2/T3/T4)	0.359	0.153–0.843	0.019
Venous system invasion (yes vs. no)	0.723	0.327–1.597	0.422
Lymphatic system invasion (yes vs. no)	1.174	0.431–3.198	0.754
Lymph node metastasis (yes vs. no)	1.289	0.522–3.179	0.582
RhoGDI2 (positive vs. negative)	0.641	0.264–1.556	0.326

RFS, relapse-free survival; pT, pathological tumor depth; RhoGDI2, Rho GDP dissociation inhibitor 2.

**Table IV.** Risk factors affecting OS rate determined by Cox proportional Hazards model in 46 patients with gastric cancer.

Variable	Multivariate analysis for OS		
	Hazard ratio	95% CI	P-value
pT (T1 vs. T2/T3/T4)	0.399	0.166–0.960	0.040
Venous system invasion (yes vs. no)	0.826	0.374–1.824	0.637
Lymphatic system invasion (yes vs. no)	1.229	0.441–3.426	0.692
Lymph node metastasis (yes vs. no)	1.188	0.453–3.117	0.726
RhoGDI2 (positive vs. negative)	0.516	0.211–1.263	0.147

OS, overall survival; pT, pathological tumor depth; RhoGDI2, Rho GDP dissociation inhibitor 2.
